# Targeting pancreatic beta cell death in type 2 diabetes by polyphenols

**DOI:** 10.3389/fendo.2022.1052317

**Published:** 2022-11-17

**Authors:** Ana García-Aguilar, Carlos Guillén

**Affiliations:** ^1^ Department of Pharmacology, Pharmacognosy and Botany, Faculty of Pharmacy, Complutense University of Madrid, Madrid, Spain; ^2^ Diabetes and Associated Metabolic Diseases Networking Biomedical Research Centre Centro de Investigación Biomédica en Red. Diabetes y Enfermedades Metabólicas asociadas (CIBERDEM), Instituto de Salud Carlos III, Madrid, Spain; ^3^ Department of Biochemistry and Molecular Biology, Faculty of Pharmacy, Complutense University of Madrid, Madrid, Spain

**Keywords:** diabetes, pancreatic beta cells, ER stress, autophagy, amylin, polyphenols

## Abstract

Diabetes is a very complex disease which is characterized by the appearance of insulin resistance that is primarily compensated by an increase in pancreatic beta cell mass, generating hyperinsulinemia. After time, pancreatic beta cells die by apoptosis appearing in the second phase of the disease, and characterized by hypoinsulinemia. There are multiple conditions that can alter pancreatic beta cell homeostasis and viability, being the most relevant ones; ER stress, cytotoxicity by amylin, mTORC1 hyperactivity, oxidative stress, mitochondrial dysfunction, inflammation and alterations in autophagy/mitophagy flux. In addition, the possible effects that different polyphenols could exert in the modulation of these mechanisms and regulating pancreatic beta cell viability are analyzed. It is necessary a profound analysis and understanding of all the possible mechanisms involved in the control and maintenance of pancreatic beta cell viability to develop more accurate and target treatments for controlling beta cell homeostasis and preventing or even reversing type 2 diabetes mellitus.

## Introduction

Type 2 diabetes mellitus (T2DM) is a very complex metabolic disease characterized by insulin resistance as well as pancreatic β cell dysfunction, and it is considered a worldwide epidemic (1). The pathogenesis of T2DM is multifactorial and a subject of continuous intense investigation. β cells are a type of cells existing in the pancreatic islets of Langerhans that secrete insulin and amylin in response to increasing glycemia. Glucose stimulates transcription, translation and exocytosis of insulin in β cells to maintain systemic glucose homeostasis. In this sense, β cells sense glucose which leads to an increase in the intracellular ATP/ADP ratio through its metabolism, thus, closing ATP-dependent potassium channels, depolarizing cellular membrane, stimulating calcium influx, and finally promoting insulin secretion.

## Effects of gluco-, lipo- and glucolipotoxicity in insulin secretion

Plasma free fatty acids (FFAs) and glucose exert both positive and negative effects on pancreatic beta cell survival and insulin secretory function, depending on their concentration and duration.

In the presence of chronic hyperglycemia (glucotoxicity), the uncoupling protein 2 (UCP2) expression in beta cells is increased, and this event is associated with a reduction in the ratio of ATP to ADP, thus inhibiting glucose-stimulated insulin secretion (GSIS), which contributes to the development of T2DM (2–4). In this regard, it was described that UCP2 upregulation, by the deletion of the deacetylase SIRT1, contributes to diminish GSIS (5). More recently, other authors demonstrated that ROS and GSIS were increased in a β-cell–specific UCP2 knockout (βUCP2KO) model, highlighting that UCP2 negatively regulates GSIS by reducing mitochondrial ROS production, and not through a defect in ATP production (6).

In the same way, a chronically exposure of β cells to elevated concentration of FFAs, referred as lipotoxicity, results in disturbances in lipid metabolism regulation, impairs GSIS by an induction in UCP2 expression, increases beta-cell apoptosis and consequently induces T2DM (7, 8).

Regarding the effects of a combined and long-term exposure to elevated glucose and FFAs (glucolipotoxicity) on pancreatic beta-cell function and survival, recently it has been demonstrated that it leads to decreased GSIS and impaired insulin gene expression, contributing to β-cell failure and T2DM (9).

## Dysfunctional mechanisms leading to beta cell dysfunction and T2DM progression

Different molecular mechanisms trigger beta cell dysfunction in a glucolipotoxic scenario and includes cytotoxicity of amylin, mTORC1 hyperactivation, ER and oxidative stress, mitochondrial dysfunction, autophagy impairment and islet inflammation. The main scope of this review is to shed light on the molecular mechanisms produced in pancreatic beta cells that lead to beta cell dysfunction, contributing to T2DM.

### ER stress

The endoplasmic reticulum (ER) of beta cells has a high capacity of protein synthesis and folding. However, in the context of insulin resistance, beta cells need to synthesize insulin beyond their capacity for protein folding and secretion, and thereby activate the unfolded protein response (UPR). UPR is an adaptive signaling pathway to promote cellular survival upon accumulation of misfolded proteins in the ER. UPR signaling sensors are inositol-requiring enzyme 1 (IRE1); PKR-like ER kinase (PERK) and the activating transcription factor 6 (ATF6). However, if UPR is chronically activated, and protein-folding demand in the ER exceeds capacity, unfolded proteins start to accumulate within the ER leading to ER stress and cell death (10, 11).

In β-cells, chronic hyperglycemia and hyperlipidemia, especially the exposure to saturated long-chain free fatty acids, known as important causative factors of T2DM, enhanced ER calcium depletion, induce ER stress and thereby cause β-cell failure (11–13).

The relationship between ER stress-induced β-cell dysfunction and death has been extensively studied (14–17) and it was first evidenced in a rare autosomal recessive form of juvenile diabetes, the Wolcott-Rallison syndrome (18). In this syndrome, mutations have been identified in the EIF2AK3 gene encoding PERK, causing a loss-of-function of this protein and promoting ER stress-mediated β-cell death (19).

Furthermore, the pathogenesis of Wolfram syndrome, an autosomal recessive neurodegenerative disorder associated with juvenile-onset diabetes mellitus, involves chronic ER stress in β-cells. This syndrome is caused by a loss-of-function of the wolfram syndrome gene 1 (WFS1), a component of the UPR that participates in maintaining ER homeostasis in β-cells (20, 21).

### Cytotoxicity of amylin

Amylin, or islet amyloid polypeptide (IAPP), is a 37-amino acid peptide hormone co-secreted with insulin by pancreatic islet β-cells in response to nutrients, including glucose, lipids or amino acids. It is a regulatory peptide that inhibits insulin and glucagon secretion in the islets, but also acts in the brain modulating satiety and inhibition of gastric emptying (22).

In human β cells, the precursor and the intermediate forms of IAPP were increased after prolonged exposure to high glucose (11). Furthermore, it is also hypothesized that amyloid aggregates composed of amylin were accumulated in the β cell of patients with T2DM, causing disruption of cell membrane (11), inflammasome activation (23), mitochondrial damage and ER-induced apoptosis (24, 25). More recently, it has been also demonstrated that overexpression of human amylin (hIAPP) in INS-1 β-cells increases the fission of mitochondria, activates mTORC1 and inhibits mitophagy, contributing to β cell death (26).

### mTORC1 hyperactivation

During the progression to T2DM, there are two phases. The first one is the insulin resistant prediabetic stage, in which the main event is the insulin resistance with normoglycemia. At this phase, β cells increase their mass by two mechanisms, to cope with an increased insulin demand: hyperplasia (cell number increase) and hypertrophy (cell size increase), with concomitant insulin and amylin secretion (27). This increase in β cell mass is accomplished by an hyperactivation of the mammalian target of rapamycin complex 1 (mTORC1) signaling, which is a key effector for the growth and survival of pancreatic β cells (28). The duration of this phase depends on the patient and, at a final stage, if mTORC1 remains chronically overactivated, pancreatic β cells fail, causing a significant reduction in β-cell mass, and thus, hypoinsulinemia appears triggering hyperglycemia. These two phases have been described in a mouse model with a chronic mTORC1 hyperactivation caused by a specific deletion of Tsc2 in β-cells (β-TSC2-/-). These mice showed an early phase with an increase in β-cell mass and an enhanced GSIS, but finally leading to β-cell failure and hyperglycemia in older mice (29). All these data pointed to mTORC1 signaling as a double-edged sword in the progression to T2DM (30).

### Altered autophagy and mitophagy

Macroautophagy, referred to here as autophagy, is a conserved and a physiological defense mechanism against acute stress that maintains cellular quality control by removing protein aggregates and damaged organelles, and acts as an essential process for maintaining cellular homeostasis in eukaryotes alternatively to the ubiquitin-proteasome system (UPS) (31, 32). Currently, there are three main types of autophagy in mammalian cells: macroautophagy, chaperone-mediated autophagy and microautophagy. Multiple signaling pathways regulate this process, but one of most important is the mTORC1 pathway, which activation induces aging and inhibits autophagy (33). Importantly, autophagy deregulation is believed to cause or contribute to aging, as well as a number of age-related diseases, including T2DM (34, 35).

It has been extensively demonstrated that autophagy protects pancreatic β cells under chronic hyperglycemia or after exposure to high fat-diet, increasing β cell survival in the progression to T2DM. In fact, specific deletion of the autophagy gene Atg7 specifically in pancreatic β cells of mice leads to impaired glucose tolerance and GSIS caused by an increase in polyubiquitinated proteins and apoptosis (36, 37). Moreover, blocking autophagy by upregulating mTORC1 signaling using β-TSC2-/- mice, increased pancreatic β cell death as a consequence of an impairment of autophagy activation as well as an induction of ER stress (29). Thus, the concept that autophagy plays a protective role in β cells is based in its capacity to alleviate oxidative (38) and ER-stress (29, 39–41), as well as to clearance hIAPP and polyubiquitin protein aggregates formed in pancreatic islets, reducing their toxicity (42, 43), and consequently, preventing pancreatic β cell failure (29, 44).

Mitophagy is a specialized type of autophagy that eliminates damaged and dysfunctional mitochondria and serves to maintain energy balance, mitochondrial quality control and cellular protection against oxidative stress. The PTEN-induced kinase 1 (PINK1)-Parkin pathway plays a major role in mediating mitophagy. PINK1 protein is a sensor of mitochondrial depolarization because it accumulates specifically on the outer mitochondrial membrane (OMM) of damaged mitochondria, and from there, it recruits Parkin to mitochondria and activates it. Furthermore, Parkin protein is an E3 ubiquitin ligase that induces ubiquitination of several OMM proteins, and consequently, activates mitophagy (45). The role of Parkin in the mitophagy of β cells has been assessed in *Parkin*-/- streptozotocin-treated mice, which showed an impairment of glucose tolerance, and a reduction in ATP content as well as in GSIS (46). Moreover, mitophagy is activated under stressful conditions, such as an exposure to proinflammatory cytokines that induce mitochondrial damage. Thus, mitophagy-deficient β cells are more vulnerable to inflammatory stress, leading to the accumulation of dysfunctional and fragmented mitochondria, increasing β-cell death and exacerbating hyperglycemia (47).

In addition, prohibitin 2 (PHB2) is an inner mitochondrial membrane (IMM) protein, very important in development and cell proliferation, but it also has a functional role as a mitophagy receptor (48, 49) as well as in maintaining mitochondrial integrity and function. Indeed, *in vivo* ablation of Phb2 specifically in β-cells (β-*Phb2*-/-) resulted in an impairment of mitochondrial function, which leads to a loss of β-cell mass and GSIS, attributed to an increased apoptosis of β-cells (50).

Interestingly, the pancreatic duodenal homeobox factor 1 (PDX1), similar to other genes that cause monogenic diabetes of the young and T2DM, is a transcription factor essential for the development of the pancreas (51–53), and was also implicated in the control of mitophagy in pancreatic β-cells (54). Indeed, PDX1 deficiency has been associated with impaired mitochondrial function and mitophagy as well as a reduced GSIS by inhibition of the nuclear encoded mitochondrial factor A (TFAM) (55). In this sense, a high expression of microRNA-765 which targets and reduces the expression of PDX1, was founded in T2DM patients and it was correlated with an inhibition of both mitochondrial respiration and β-cell function (56).

Furthermore, mitochondrial dynamics (fission/fusion) are essential to maintain a balance between mitochondrial biogenesis and mitochondrial turnover. Recently, there is an increasing interest in understanding the role of mitochondrial dynamics in the development of T2DM. Regarding this, an imbalance between these processes leads to a reduction in mitophagy and an accumulation of dysfunctional mitochondria (57–59). Mitochondrial fusion is mainly coordinated by three GTPases, the homologous mitofusins 1 and 2 (MFN1, MFN2) localized in the OMM, and the optic atrophy 1 (OPA1) protein, which resides in the IMM. In contrast, fission is regulated by the soluble GTPase dynamin-related protein 1 (Drp1) and the mitochondrial fission 1 protein (FIS1). Herein, it is interesting to note that both FIS1 overexpression and FIS1 knockdown lead to a decrease in GSIS (60, 61), suggesting that GSIS in beta cells requires a precise expression level of this fission protein (62). Accordingly, a downregulation of Drp1 in the pancreatic β cell line INS-1E as well as in spread mouse islets significantly reduced the expression of mitochondrial fusion proteins (MFN1, MFN2 and OPA1), downregulated ATP content and GSIS (63). Additionally, an increase in the mRNA and protein expression of MFN2 and several mitophagy-related proteins (NIX, PINK1, and PARKIN) has been reported in prediabetic subjects, whereas patients with T2DM showed a decreased expression of these proteins (64), demonstrating the important role of mitophagy and mitochondrial dynamic in the pathogenesis of T2DM.

### ROS and oxidative stress

The role of reactive oxygen species (ROS) in β cell function depends on the timing and strength of the signal. Moreover, β cells are more vulnerable to oxidative stress because of their minor capacity of scavenging oxidants when compared to other types of cells (65). Herein, a transient and moderate production of mitochondrial reactive oxygen species (mROS) is an important signaling to promote β cell function and GSIS, mimicking the glucose effect (6, 66). However, a chronic and persistent elevation of ROS, as a result from inflammation or excessive glucose and fatty acid concentrations, impairs β-cell function by repressing the ROS signal and/or inducing mitochondrial damage that also results in an increase in ROS production (67, 68). This increase in mitochondrial superoxide production activates UCPs *via* peroxidation of mitochondrial phospholipids. In agreement with this, morphology studies showed that β-cells from patients with T2D and from non-diabetic donors had similar numbers of mitochondria, but the mitochondrial density volume was significantly higher in diabetic islets, which has been associated with an increase in ROS production. In line with this, an upregulation of UCP2 protein levels was observed in type 2 diabetic islets when compared to non-diabetic ones (3).

Furthermore, ROS are able to oxidize cardiolipin (CL) and other mitochondrial inner membrane phospholipids, initiating the permeabilization of the outer mitochondrial membrane and subsequent release of cytochrome c into the cytosol, triggering apoptosis and β-cell mass reduction (69, 70).

Also, the group VIA Ca^2+^-independent phospholipase A2 (iPLA_2_ *β*) proteins is attracting increasing interest as a central participant in CL remodeling and protects β cell mitochondria from oxidative damage (71). In fact, mutations in tafazzin (TAZ), a mitochondrial phospholipid-lysophospholipid transacylase that participates in CL remodeling, are implicated in Barth syndrome, in which patients lacking functional TAZ present with cardiomyopathy and skeletal dysfunction due to a total CL deficiency. Very recently, it has been demonstrated a reduced *ex vivo* insulin secretion under non-stimulatory low-glucose concentrations in islets isolated from TAZ KD mice, highlighting the importance of TAZ in regulating normal β-cell function (72).

### Mitochondrial dysfunction

Mitochondrial DNA (mtDNA) mutations and chronic metabolic changes, such as glucotoxicity, are the main mechanisms that contribute to β cell mitochondrial dysfunction that results in an enhanced β cell apoptosis in T2DM (73).

Among mtDNA mutations, a reduced expression in the mitochondrial transcription factor B1 (TFB1M) of pancreatic islets founded in a β-cell specific KO of TFB1M (β-*Tfb1m*-/-) or caused by a variant of the *TFB1M* gene (rs950994), displayed mitochondrial dysfunction, reduced ATP production and, consequently, impaired GSIS (74, 75). Furthermore, there were an increase in the percentage of mitochondria with vesicular and swollen morphology, as long as an impairment of autophagy and mitophagy flux in β cells from β-*Tfb1m*-/- mice when compared to control islets (76). Together, these results highlighted the important role of TFB1M in mitochondrial and cellular function in pancreatic β cells. According to this, mitochondrial fragmentation occurs in β cells exposed to high-fat diet (HFD) (77).

In view of metabolic changes, hyperglycemia, a hallmark of T2DM, promotes the transfer of reducing equivalents to the respiratory chain in mitochondria of pancreatic β cells, resulting in the hyperpolarization of the mitochondrial membrane potential (ΔΨM) and generation of ATP. Compared to control islets, diabetic islets display a decreased expression of several proteins involved in oxidative metabolism, including several components of the mitochondrial respiratory chain (78). Moreover, diabetic islets showed reduced hyperpolarization of the ΔΨM, lower ATP levels at high glucose, impaired Ca2+ signaling and lowered GSIS (78–80). In this sense, it has been determined very recently that a reduction in the activity of the cytochrome C oxidase (complex IV) in islets could be a primary inborn defect that underlies β cell dysfunction (81).

### Islet inflammation

Inflammatory stress plays a crucial role in the pathogenesis of T2DM. In fact, a mild inflammation inside islets can be detected in T2DM patients. In this regard, the administration of anti-inflammatory drugs generates a mild glucose decrease, suggesting that inflammation is involved in T2DM, although it is still unknown whether the effect of these anti-inflammatory medications has a direct impact in pancreatic islets or it is through the effect in other relevant metabolic tissues. In fact, this inflammation could be derived from nutrient overload, proinflammatory cytokines, amylin accumulation or ER stress (82). One of the organelles that is found altered after inflammation is mitochondria, with a dysfunctional production of ATP and inducing the proapoptotic machinery. An important mechanism to combat the accumulation of altered mitochondria after an inflammatory process is mitophagy, which is the specific mitochondrial clearance by using the autophagic machinery. In this regard, any change in mitophagy sensitizes pancreatic beta cells to inflammation. Then, mitophagy is a survival mechanism in these cells in response to inflammation (47). Oxidative stress is able to affect mitochondria and generates a higher amount of ROS because of the impairment in the antioxidant defenses, leading to mitochondrial dysfunction and pancreatic beta cell failure (83).

## Effects of toxic metabolites in β cell function and viability

Apart from all the mechanisms previously explained having a deleterious effect on pancreatic beta cells’ viability, there are several toxic metabolites coming from the environment such as pollutants or certain treatments that lead to a decrease in beta cell survival. Among the most relevant metabolites are the following: bisphenol A (BPA), heavy metal exposure, glucocorticoids and dioxins. BPA is a common contaminant found in the environment and it has been related with both T1DM and T2DM because of its involvement in the reduction and impairment of pancreatic β cells (84, 85). BPA is known as an endocrine disruptor, affecting the hormonal system and, as it was mentioned before, modifying cellular metabolism. Very importantly, BPA not only affects pancreatic β-cells but can modify glucagon secretion produced by α cells because of a switch in β to α cell ratio transition. Then, BPA is considered to affect the endocrine pancreas (86, 87). BPA has a lipophilic nature and most of the receptors are intracellular including binding to specific hormonal receptors such as sex hormone receptors for estrogen (ER) and androgen (AR) as well as thyroid hormone receptor (TR) and glucocorticoid receptor (GR). However, BPA can bind to other receptors located in the membrane such as the membrane estrogen receptor (mER) and G protein-coupled receptor (GPR30) (88). Very importantly, pancreatic β cell function is mediated by ERα and ERβ in response to BPA altering the expression and function of different ion channel (89).

Another important group of toxic metabolites is the exposition to heavy metals including cadmium, zinc, inorganic arsenite, manganese affecting to glucose homeostasis increasing the risk to suffer diabetes (90). For instance, the effect of cadmium is through an accumulation of lipids and an increase in both inflammation and in insulin secretion (91) and inducing the activation of c-Jun N-terminal kinases (JNK) and apoptosis (92). Arsenite is another toxic metabolite that is even more toxic than cadmium or manganese, because of its capacity to regulate miR-146A, controlled by nuclear factor-kB (NF-kB). In addition, arsenite downregulates the calcium-dependent protein kinase (CAMK2a), which regulates insulin secretion (93). Glucocorticoids are a group of steroids which are secreted by the adrenal cortex in our body and it is involved in the degradation and mobilization of stored energy involved in tissue repair, metabolic processes and many other functions. Glucocorticoids facilitate the appearance of insulin resistance and diabetes (94, 95). Furthermore, dioxins are other group of pollutants generates pancreatic β cell dysfunction (96). There are several mechanisms involved in pancreatic β cell toxicity produced by dioxins that have been reviewed very recently (97).

## Prevention of beta cell dysfunction by polyphenols

Although there are multiple types of natural products, polyphenols represent more than 8000 compounds found in plants, presenting high evidence of its protective role in different metabolic and neurodegenerative diseases (98, 99). Polyphenols are a group of natural products that exert their antidiabetic effects *via* a variety of mechanisms which have been extensively studied and include an improvement in mitochondrial function by scavenging ROS which reduces oxidative damage. These treatments have in common an increase in mitochondrial function and biogenesis by the modulation of several pathways, including AMPK, SIRT1 and NRF-1 targets. One of the mechanisms involved in the protection of these compounds is the enhancement of autophagic activity, alleviating the ER stress and mitochondrial dysfunction provoked by the accumulation of misfolded proteins in these cells (100). Polyphenols represent a promising chemical group for the prevention and treatment of several metabolic diseases such as T2DM. But, these compounds have additional properties such as regenerative capacity. This is important since, apart from avoiding its disappearance, it is possible to mediate an increase in the number of cells, having supplemental beneficial consequences (101).

As it was mentioned before, mTORC1 hyperactivation is a key event in the progression to T2DM. There is abundant evidence that important mechanisms involved in the protective effects of these polyphenols against the decline of β-cells have also been linked to autophagy enhancement, related to mTORC1 inhibition, with or without implication of AMPK activation. Then, mTORC1 downregulation through AMPK-dependent and AMPK-independent mechanisms are essential in the survival of pancreatic β cells. Different natural products have been involved in its modulation and hence, regulating the protective effect of autophagy including resveratrol, curcumin, ECCG, punicalagin, oleuropein and many others. Although many compounds have been studied, one of the best characterized molecules is resveratrol by the use of a great variety of approaches and very recently reviewed in (98). Regarding *in vitro* studies, resveratrol inhibits mTORC1 signaling pathway by the modulation of the acetylation status of TSC2 (102). Very recently this mechanism has been corroborated *in vivo*, avoiding lipid accumulation in the liver combating obesity and complications of diabetes (103). Autophagy promotes β-cell survival by enabling adaptive responses to alleviate ER stress, mitochondrial dysfunction and oxidative stress. In fact, there are many papers suggesting that mitophagy enhancement is a potential mechanism to preserve β-cell function and delay the progression of T2DM (104). Another important molecule involved in pancreatic β cell failure in T2DM is human amylin (hIAPP). hIAPP is a protein co-secreted with insulin by pancreatic β cells and possesses a higher propensity to misfold and aggregate inside these cells. This propensity is especially higher when there is an increase in insulin synthesis demand, which occurs during the progression to T2DM, a long period of time with a characteristic insulin resistance and an mTORC1 hyperactivity (28). Under these conditions, autophagy activation has protective effects and inhibits pancreatic β cell death, being a natural defense (42, 43). Flavonoids have also been involved in the protection of the deleterious effects of hIAPP aggregates in these cells (105–109). Apart from the actions of flavonoids already explained, flavonoids have been also involved in an increase in antioxidant enzymes, as a protective mechanism (110, 111). Very importantly, flavonoids are also important regulators of the aging process theyself, affecting not only diabetes but different age-associated diseases (112).

**Figure 1:**
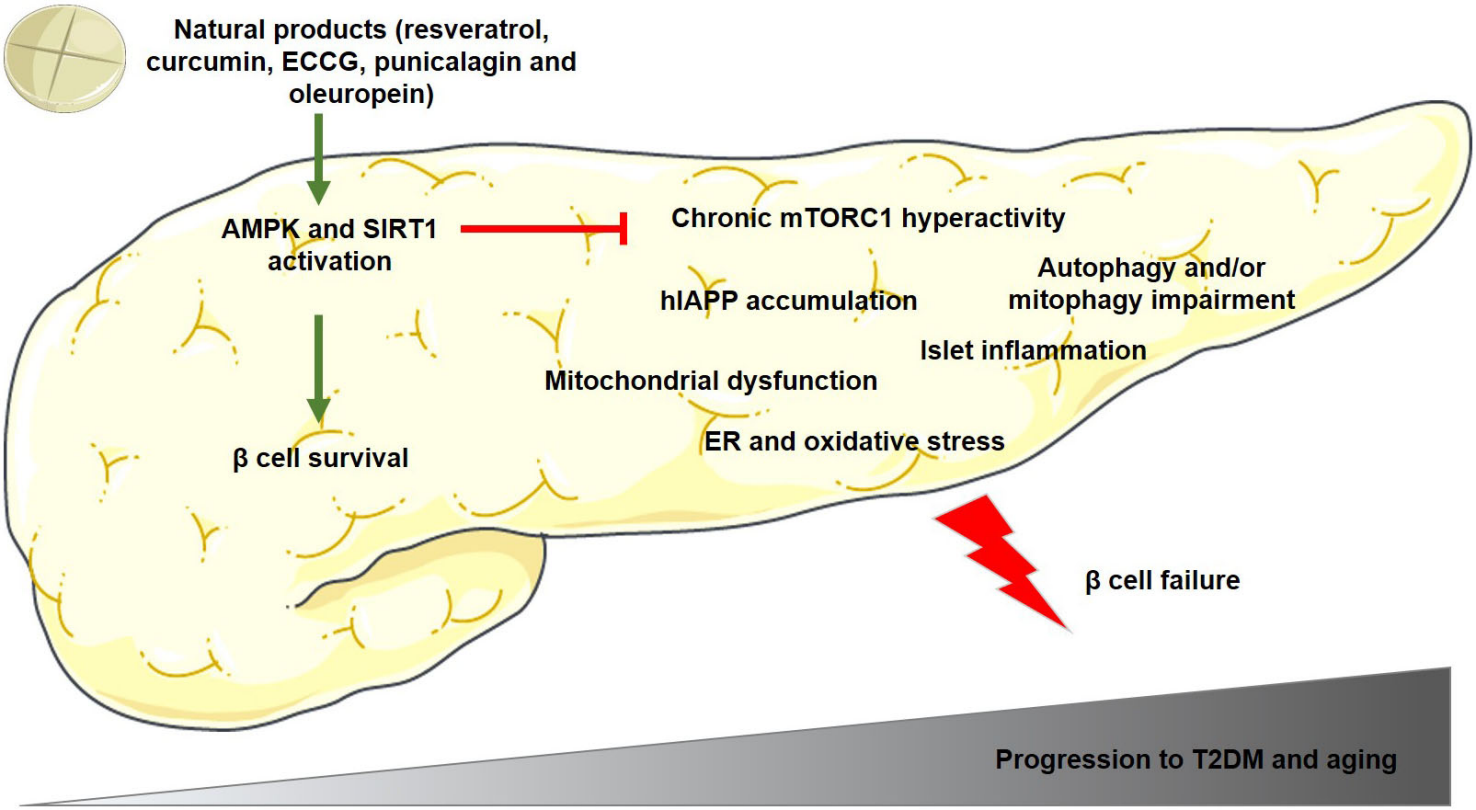
Main mechanisms involved in pancreatic β cell dysfunction and apoptosis during the progression to T2DM and/or aging. The polyphenols mentioned in this figure have been demonstrated to activate AMPK and SIRT1 proteins in β cells, which reduces mTORC1 hyperactivation and β cell failure, and as a consequence, preventing the onset and progression of T2DM. Green arrows indicate activation and red lines indicate inhibition of the activity of the target protein. AMPK, adenosine monophosphate (AMP)-activated protein kinase; ECCG, Epigallocatechin gallate; ER, endoplasmic reticulum; human amylin, hIAPP; mTORC1, mammalian/mechanistic target of rapamycin complex 1; SIRT1, sirtuin-1; T2DM, type 2 diabetes mellitus.

Altogether, there are several molecular mechanisms involved in β cell dysfunction and apoptosis, being some of the most important those depicted in **Figure 1**. This figure also shows the beneficial effects of some polyphenols in pancreatic β cells, increasing cell survival and preventing the onset and progression of T2DM.

## Conclusion

In this review, the main contributors to pancreatic beta cell failure as well as the key protective mechanisms of different polyphenols that interfere with the different mediators of pancreatic beta cell dysfunction in the progression to T2DM have been summarized. Although many distinct signaling pathways have been described, it is essential a better and more profound understanding of the pathophysiology of the disease in order to obtain better treatments to maintain and protect pancreatic beta cells for longer periods of time, prolonging its lifespan and avoiding the appearance of diabetes.

## Author contributions

Both AG-A and CG have contributed to the writing as well as to the discussion of the manuscript. All authors contributed to the article and approved the submitted version.

## Funding

This work was supported by grants PID2020-113361RB-I00 from Ministerio de Ciencia e Innovación and CIBER de Diabetes y Enfermedades Metabólicas Asociadas, Instituto de Salud Carlos III (Spain), to CG.

## Conflict of interest

The authors declare that the research was conducted in the absence of any commercial or financial relationships that could be construed as a potential conflict of interest.

## Publisher’s note

All claims expressed in this article are solely those of the authors and do not necessarily represent those of their affiliated organizations, or those of the publisher, the editors and the reviewers. Any product that may be evaluated in this article, or claim that may be made by its manufacturer, is not guaranteed or endorsed by the publisher.
